# Supplementary material for: Mapping autophagosome contents identifies interleukin-7 receptor-α as a key cargo modulating CD4+ T cell proliferation
Source: Nat Commun. 2022 Sep 2;13:5174. doi: 10.1038/s41467-022-32718-x (PMC9440129; doi:10.1038/s41467-022-32718-x)
Supplement: Supplementary file 1 — Supplementary Information [file 41467_2022_32718_MOESM1_ESM.pdf]

Supplementary Materials for

Mapping autophagosome contents identifies interleukin-7 receptor-α as a key cargo modulating CD4+ T cell proliferation

Dingxi Zhou[1], Mariana Borsa[1], Daniel J. Puleston[1], Susanne Zellner[2], Jesusa Capera[1], Sharon Sanderson[1], Martina Schifferer[3,4], Svenja S. Hester[5], Xin Ge[1], Roman Fischer[5,6], Luke Jostins[1], Christian Behrends[2#], Ghada Alsaleh[1,7#], Anna Katharina Simon[1#*]

[#]Authors contributed equally

*Corresponding author: katja.simon@imm.ox.ac.uk

Supplementary Data 2

Supplementary Fig. 1 Autophagy-deficient CD4+ T cells show delayed proliferation. 4

Supplementary Fig. 2 Validation of the *Lc3b-AP2* transgenic mouse model. 7

Supplementary Fig. 3 IL-7Rα is an autophagosomal cargo in CD4+ T cells. 9

Supplementary Fig. 4 Impaired IL-2R assembly in activated T-Atg7$^{-/-}$ CD4+ T cells. 11

Supplementary Table 1. The sequences of the primers that were used in this research...13

Supplementary Data

Supplementary Fig. 1

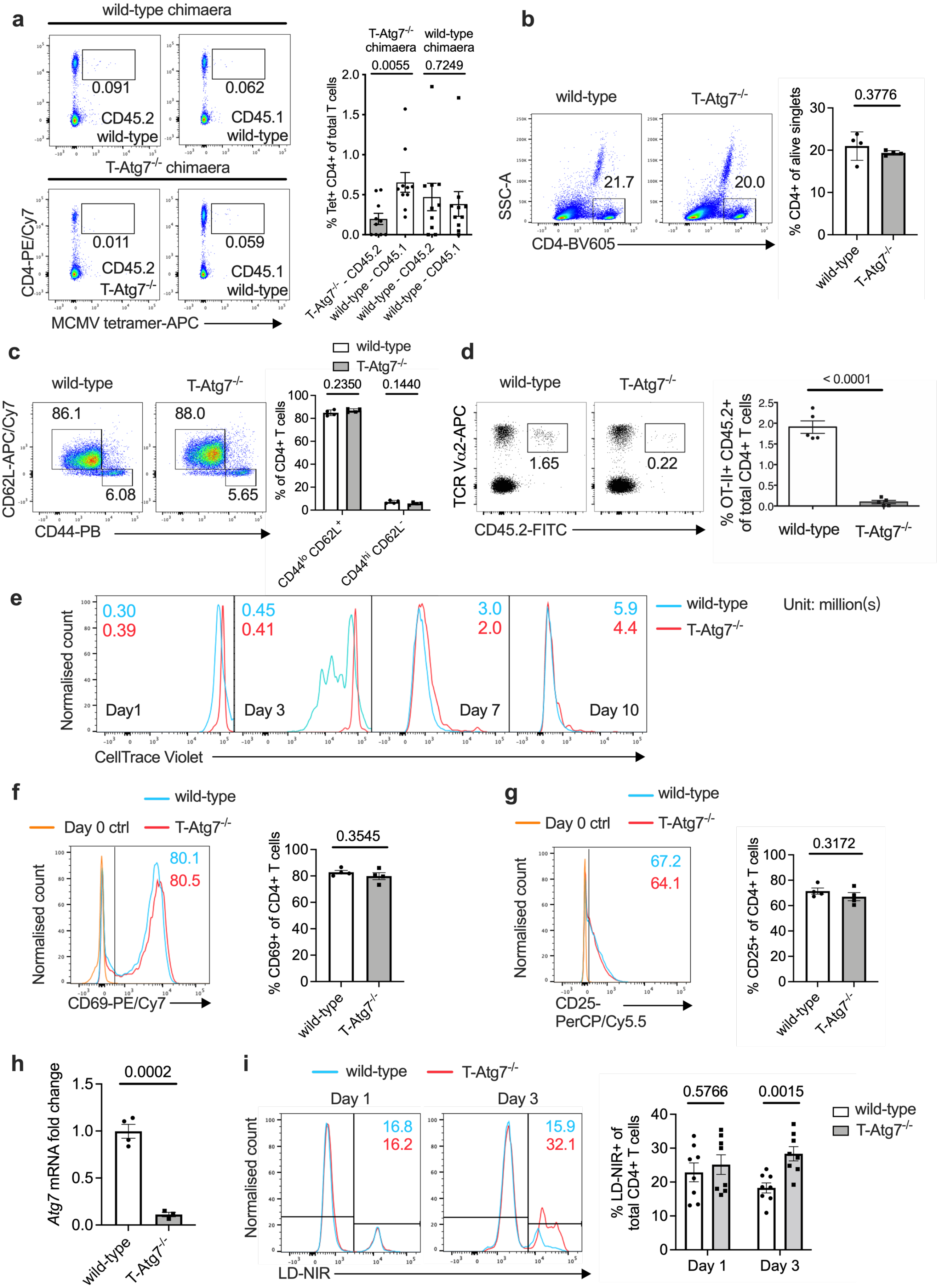

Supplementary Fig. 1 Autophagy-deficient CD4+ T cells show delayed proliferation.

(a) Dot plots (left) of murine cytomegalovirus (MCMV)-specific CD4+ T cells among CD19- population from lungs of CD45.2+ wild-type or T-Atg7$^{-/-}$ donors and CD45.1+ donors. Bar graph (right) indicates the percentage of Tet (tetramer)+ CD4+ T cells within CD45.2+ CD19- or CD45.1+ CD19- population (n = 9 mice per group) as mean ± SEM with paired two-tailed Student's t test.

(b, left) and **(c, left)** show dot plots of splenic CD4+ T cells, and CD44^{hi} CD62L^{-} T cells within total CD4+ T cells, in 6–8-week wild-type or T-Atg7$^{-/-}$ OT-II mice. Bar graphs **(b and c, right)** indicate the percentages of gated populations within total splenocytes (n = 4 mice per group).

(d) Dot plots (left) are gated on CD45.2+, TCR Vα2+ in the blood of recipient mice. Bar graph (right) depicts the frequency of gated population within total CD4^{+} T cell population (n = 5 mice per group).

(e) Splenic OT-II+ T cells from wild-type or T-Atg7$^{-/-}$ mice were stimulated with ovalbumin for 1, 3, 7 or 10 days in vitro. Histograms represent OT-II+ CD4+ T cell proliferation. Number indicates the total cell number of gated population in million(s).

(f, left) and **(g, left)** show histograms of CD69+ and CD25+ population within CD4+ T cells on day 1 of activation in vitro. Bar graphs **(f and g, right)** indicate the percentages of gated populations within live CD4+ population (n = 4 mice per group).

(h) Quantification of *Atg7* mRNA level in flow cytometry-sorted most divided splenic CD4+ T cells activated for 7 days (gating strategy in Figure 1e, day 7) by qRT-PCR. Bar graph shows fold change in *Atg7* mRNA of T-Atg7$^{-/-}$ normalised to wild-type (n = 3 mice for wild-type group, n = 4 mice for T-Atg7$^{-/-}$ group) as mean ± SEM with unpaired two-tailed Student's t test.

(i, left) show histograms of LIVE/DEAD Fixable Near-IR (LD-NIR)+ population within CD4+ T cells, on day 1 and 3 of activation in vitro. Bar graph **(i, right)** indicates the percentage of gated population within live CD4+ population (*n* = 8 mice per group).

All data except for (a) are represented as mean ± SEM with unpaired two-tailed Student's *t* test. Exact *p* values are depicted in the figure. All experiments are representative of three independent experiments. Source data are provided as a Source Data file.

Supplementary Fig. 2

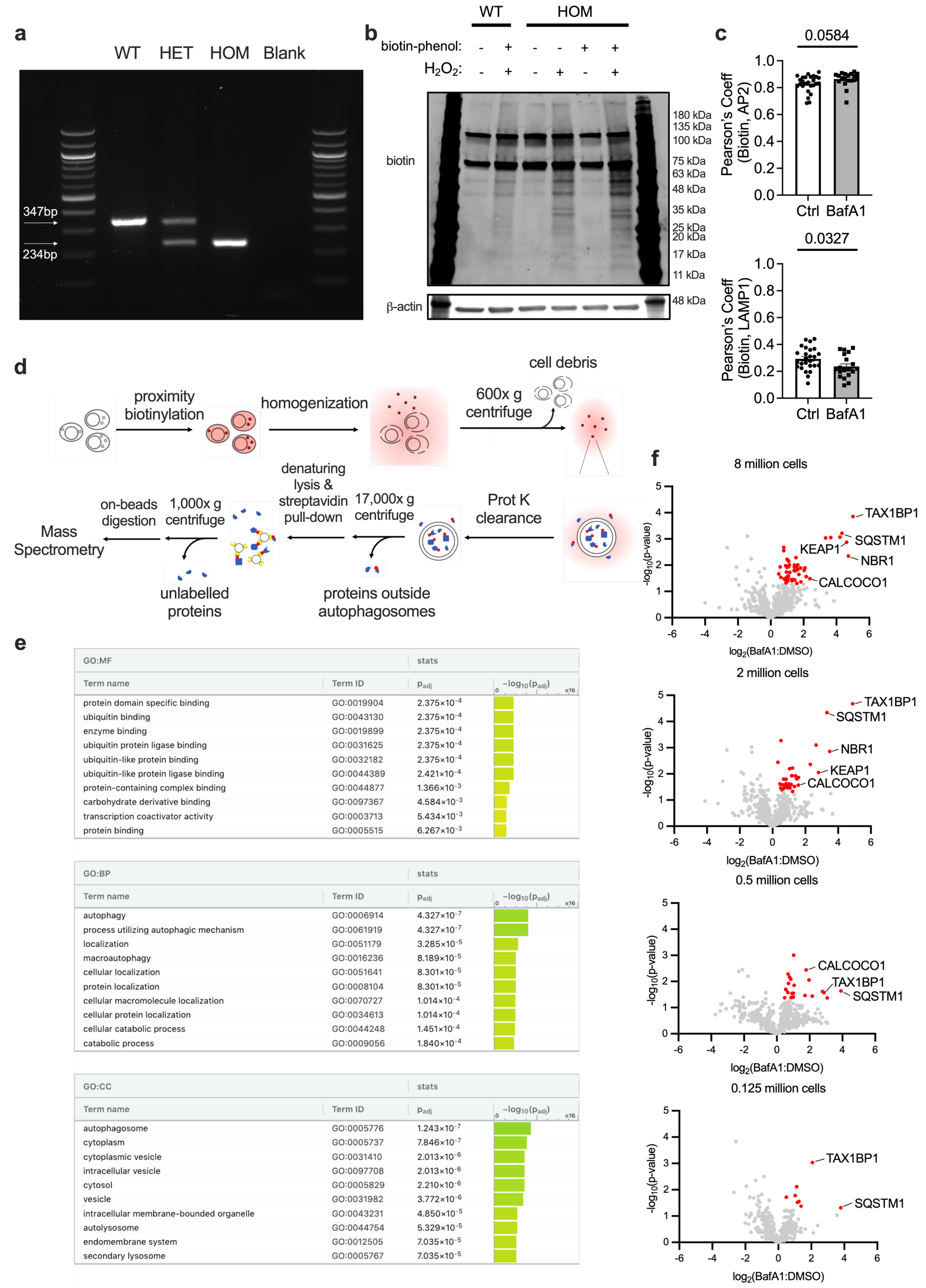

e

GO:MF

Term name	Term ID	p_{adj}
protein domain specific binding	GO:0019904	2.375×10^{-4}
ubiquitin binding	GO:0043130	2.375×10^{-4}
enzyme binding	GO:0019899	2.375×10^{-4}
ubiquitin protein ligase binding	GO:0031625	2.375×10^{-4}
ubiquitin-like protein binding	GO:0032182	2.375×10^{-4}
ubiquitin-like protein ligase binding	GO:0044389	2.421×10^{-4}
protein-containing complex binding	GO:0044877	1.366×10^{-3}
carbohydrate derivative binding	GO:0097367	4.584×10^{-3}
transcription coactivator activity	GO:0003713	5.434×10^{-3}
protein binding	GO:0005515	6.267×10^{-3}

GO:BP

Term name	Term ID	p_{adj}
autophagy	GO:0006914	4.327×10^{-7}
process utilizing autophagic mechanism	GO:0061919	4.327×10^{-7}
localization	GO:0051179	3.285×10^{-5}
macroautophagy	GO:0016236	8.189×10^{-5}
cellular localization	GO:0051641	8.301×10^{-5}
protein localization	GO:0008104	8.301×10^{-5}
cellular macromolecule localization	GO:0070727	1.014×10^{-4}
cellular protein localization	GO:0034613	1.014×10^{-4}
cellular catabolic process	GO:0044248	1.451×10^{-4}
catabolic process	GO:0009056	1.840×10^{-4}

GO:CC

Term name	Term ID	p_{adj}
autophagosome	GO:0005776	1.243×10^{-7}
cytoplasm	GO:0005737	7.846×10^{-7}
cytoplasmic vesicle	GO:0031410	2.013×10^{-6}
intracellular vesicle	GO:0097708	2.013×10^{-6}
cytosol	GO:0005829	2.210×10^{-6}
vesicle	GO:0031982	3.772×10^{-6}
intracellular membrane-bounded organelle	GO:0043231	4.850×10^{-5}
autolysosome	GO:0044754	5.329×10^{-5}
endomembrane system	GO:0012505	7.035×10^{-5}
secondary lysosome	GO:0005767	7.035×10^{-5}

Supplementary Fig. 2 Validation of the *Lc3b-AP2* transgenic mouse model.

(a) PCR genotyping of the *Lc3b-AP2* mouse model (WT, wild-type; HET, heterozygote; HOM, homozygote).

(b) Biotinylated protein profiles of whole-cell homogenates from splenocytes of wild-type (WT) or homozygotes (HOM), activated with 10 μg/mL lipopolysaccharides for 3 days. Cells were left untreated or labelled with biotin-phenol and/or H_2O_2. These experiments were repeated as biological triplicates with similar results.

(c) Bar graphs show Pearson's correlation coefficient between Biotin and APEX2 (AP2) (upper), as well as Biotin and LAMP1 (lower). Data are represented as mean ± SEM, with unpaired two-tailed Student's *t* test, n = 26 cells for Ctrl group, n = 19 cells for bafilomycin A1 (BafA1) group. Exact *p* values are depicted in the figure.

(d) Schematic overview of the procedure of purifying autophagosomal biotinylated proteins.

(e) Gene Ontology (GO) enrichment analysis of BafA1-upregulated proteins with the top terms for molecular function (MF), biological pathway (BP) and cellular compartment (CC). p_{adj} are *p* values adjusted for multiple testing using the Benjamini-Hochberg method.

(f) Volcano plots of proteins labelled by APEX2-LC3B in immortalised mouse embryonic fibroblast cells, with the titration of different cell numbers. Proteins significantly upregulated in BafA1-treated samples are highlighted in red ($p < 0.05$ by unpaired two-tail Student's *t* test, n = 3 biological replicates per group).

Source data are provided as a Source Data file.

Supplementary Fig. 3

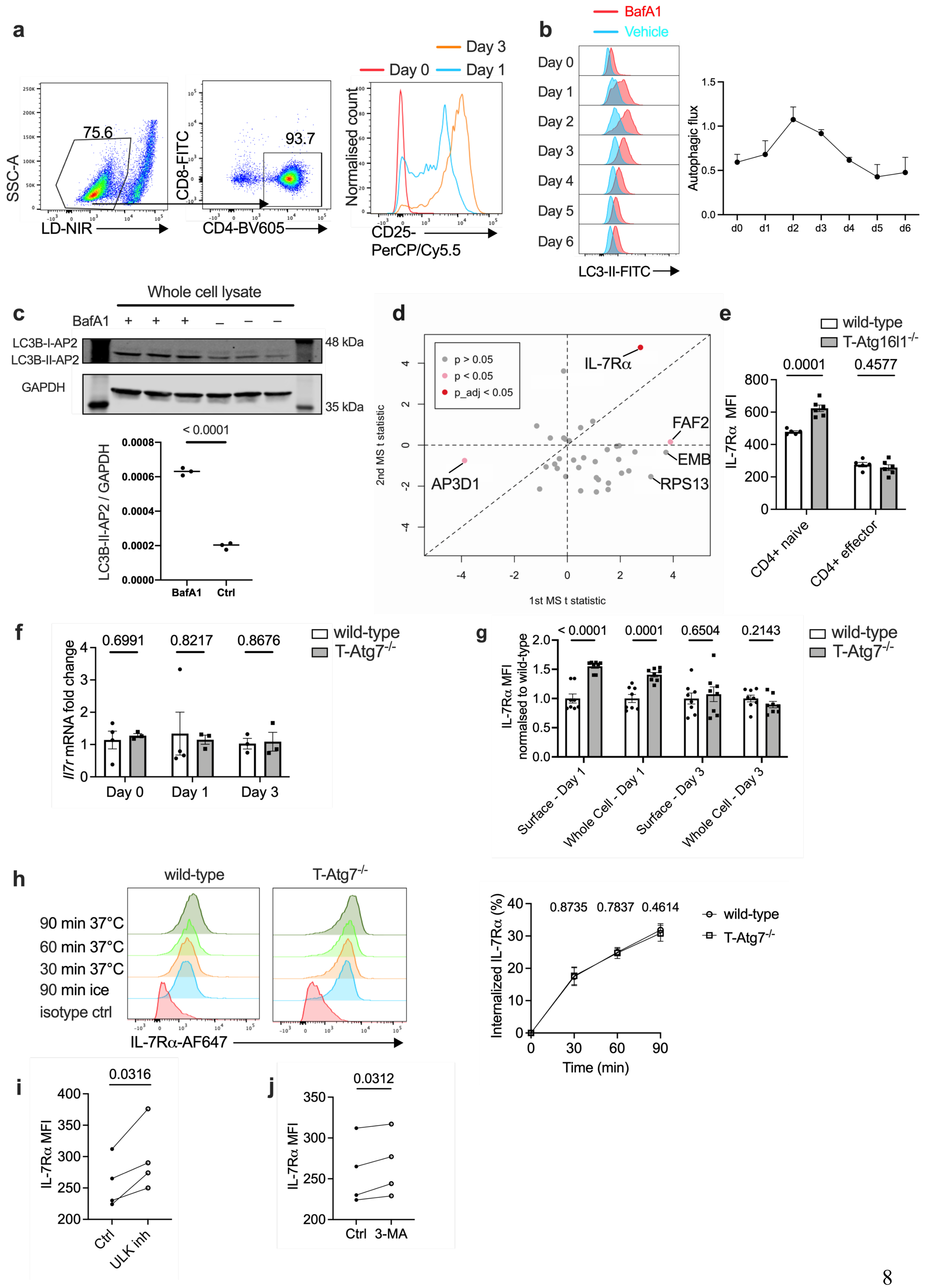

Supplementary Fig. 3 IL-7Rα is an autophagosomal cargo in CD4+ T cells.

(a) Dot plots (left) displaying the survival rate and the purity of CD4+ T cells 3 days post-activation. Histogram (right) depicts the surface level of CD25 gated on live CD4+ T cells on days 0, 1 and 3 of activation.

(b) LC3-II histograms (left) of activated CD4+ T cells on corresponding days, which were treated or not with BafA1 for 2 hours before staining. Autophagic flux (right) was calculated by the LC3-II geometric mean fluorescent intensity (BafA1 - Vehicle)/Vehicle, n = 3 mice. Data are represented as mean ± SEM.

(c) Western blot (upper) and quantitative analysis (lower) of LC3B-AP2 expression level in activated CD4+ T cells on day 3 post-activation, n = 3 mice per group.

(d) Meta-analysis of 36 proteins detected in both proximity-biotinylation experiments in 3-day-activated CD4+ T cells. Pink dots indicate the proteins upregulated upon BafA1-treatment with nominal $p < 0.05$, whereas red dot indicates the protein with $p < 0.05$ after Benjamini-Hochberg correction.

(e) Bar graph depicting surface IL-7Rα level as mean fluorescent intensity (MFI) in different CD4+ subtypes from wild-type and CD4^{cre} *Atg16l1*$^{fl/fl}$ (T-Atg16l1$^{-/-}$) mice, n = 5 mice for Ctrl group, n = 6 mice for T-Atg16l1$^{-/-}$ group.

(f) Quantification of *Il7ra* mRNA expression in splenic CD4+ T cells without activation, activated for 1 day or 3 days. Bar graph shows the fold change in *Il7ra* mRNA of T-Atg7$^{-/-}$ normalised to wild-type, n = 4 mice on day 1 and 2 for wild-type group, n = 3 mice on day 1 and 2 for T-Atg7$^{-/-}$ group, n = 3 mice on day 3 for both groups.

(g) Bar graph summarising flow results of both surface and whole-cell IL-7Rα level in CD4+ T cells activated for 1 or 3 days in vitro. Fold change in IL-7Rα geometric mean fluorescence intensity of T-Atg7$^{-/-}$ normalised to wild-type is shown, n = 8 mice per group.

(h) Internalisation of surface IL-7Rα on naïve CD4+ T cells was performed with a biotin-based flow cytometric endocytosis assay. Histograms (left) show biotin-conjugated IL-7Rα antibody level. Connected scatter plot (right) indicates the percentage of internalised IL-7Rα at 30, 60, and 90 min at 37 °C, n = 6 mice per group.

(i) and **(j)** Levels of IL-7Rα gated on naïve CD4+ T cells from wild-type mice, treated or not with 2.5 μM SBI-0206965 (i, ULK inh) or 10 μM 3-methyladenine (j, 3-MA) for 20 hours. Statistics by paired two-tailed Student's t test, n = 4 mice per group.

All values represented as mean ± SEM with unpaired two-tailed Student's t test unless being further addressed. Exact p values are depicted in the figure. Quantitative analyses are representative of three independent experiments with similar results. Source data are provided as a Source Data file.

Supplementary Fig. 4

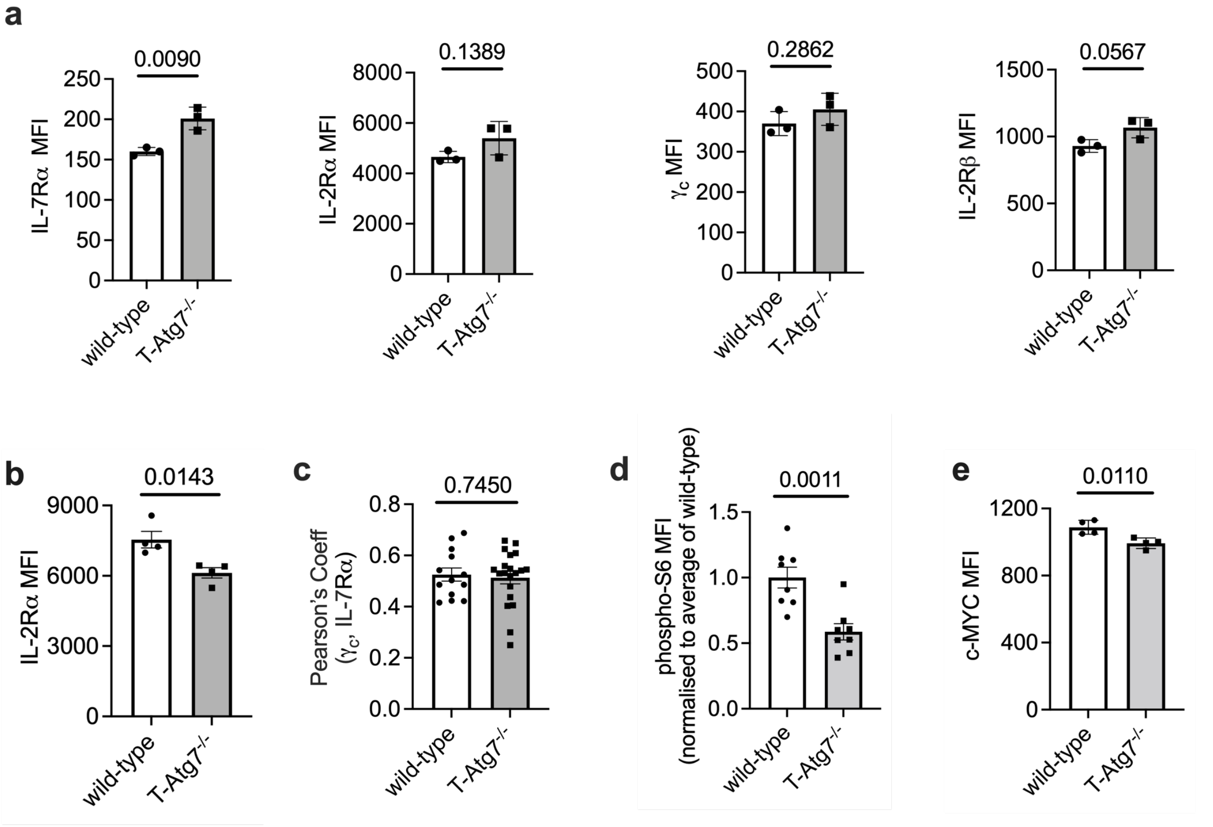

Supplementary Fig. 4 Impaired IL-2R assembly in activated T-Atg7$^{-/-}$ CD4+ T cells.

(a) Splenic naïve CD4+ T cells purified from either wild-type or T-Atg7$^{-/-}$ mice were activated with anti-CD3/CD28 Dynabeads for 24 hours. The surface receptor levels were assessed with flow cytometry. Bar graphs depict surface levels of IL-7Rα, IL-2Rα, common gamma chain (γ_c) and IL-2Rβ on activated CD25+ CD4+ T cells, *n* = 3 mice per group.

(b) Bar graph of CD25 surface level, gated on activated CD4+ T cells activated for 3 days in vitro, *n* = 4 mice per group.

(c) Naive CD4+ T cells were pre-treated with anti-CD3/CD28 Dynabeads for 24 hours to upregulate IL-2Rα. Then, cells were exposed to supported lipid bilayers containing ICAM1 and anti-CD3 proteins and fixed after a 10-min incubation in the presence of 50 U ml^{-1} of murine IL-2. Synapse formation was confirmed by the presence of an ICAM1 ring.

Cytokine receptors were stained for TIRF imaging. Bar graph shows Pearson's correlation coefficient of the co-localisation of IL-7Rα and γ_c at the mature immune synapses of activated CD25+ CD4+ T cells, which were exposed to lipid bilayers and incubated with murine IL-2, *n* = 13 cells for wild-type group, *n* = 20 cells for T-Atg7$^{-/-}$ group.

(d) Bar graph shows the fold change in phospho-S6 geometric mean fluorescence intensity of CD25+ CD4+ T cells from T-Atg7$^{-/-}$ mice normalised to the ones from wild-type mice, *n* = 8 mice per group.

(e) Bar graph shows the c-MYC geometric mean fluorescence intensity of CD25+ CD4+ T cells activated for 1 day, from both wild-type and T-Atg7$^{-/-}$ mice, *n* = 4 mice per group.

All data are represented as mean ± SEM with unpaired two-tailed Student's *t* test. Exact *p* values are depicted in the figure. Quantitative analyses are representative of three independent experiments with similar results. Source data are provided as a Source Data file.

Supplementary Table 1. The sequences of the primers that were used in this research.

**Genes**	**Primer**
CD4^{cre} Fwd	5'-CGATGCAACGAGTGATGAGG-3'
CD4^{cre} Rev	5'-GCATTGCTGTCACTTGGTCGT-3'
Atg7 WT Fwd	5'-CCATGCTGATGGCTAATGTCTC-3'
Atg7^{flox} Fwd	5'-CTGCAGGAATTCGATATCATAACTTCG-3'
Atg7 Rev	5'-GTCCAGAGTCCGGTCTCTGGTTG-3'
Lc3b-APEX2 Fwd	5'-GTGGGGAACCTCTCAACTGTCAG-3'
Lc3b-APEX2 Rev	5'-GTGGGGAACCTCTCAACTGTCAG-3'
Atg16l1^{flox} Fwd	5'-CTTTCTCAACAGAACCAGCAGTAC-3'
Atg16l1^{flox} Rev	5'-GTAGAAAGACTGGTGATGGTAAACC-3'
