# Supplementary material for: Mapping autophagosome contents identifies interleukin-7 receptor-α as a key cargo modulating CD4+ T cell proliferation
Source: Nat Commun. 2022 Sep 2;13:5174. doi: 10.1038/s41467-022-32718-x (PMC9440129; doi:10.1038/s41467-022-32718-x)
Supplement: Supplementary file 3 — Description of Additional Supplementary Files [file 41467_2022_32718_MOESM3_ESM.pdf]

Description of Additional Supplementary Files

Supplementary Data 1. Proteinase K-protected and streptavidin-enriched proteins, using *Lc3B-AP2* imMEF cells treated with BafA1 or DMSO.

Supplementary Data 2. Proteinase K-protected and streptavidin-enriched proteins, using *Lc3B-AP2* CD4+ T cells activated with anti-CD3/CD28 beads for 3 days and treated with BafA1 or DMSO.

Supplementary Data 3. Meta-analysis combining proteinase K-protected and streptavidin-enriched proteins from two experiments, using *Lc3B-AP2* CD4+ T cells activated with anti-CD3/CD28 beads for 3 days and treated with BafA1 or DMSO.
