# Supplementary material for: Mapping autophagosome contents identifies interleukin-7 receptor-α as a key cargo modulating CD4+ T cell proliferation
Source: Nat Commun. 2022 Sep 2;13:5174. doi: 10.1038/s41467-022-32718-x (PMC9440129; doi:10.1038/s41467-022-32718-x)
Supplement: Supplementary file 7 — Reporting Summary [file 41467_2022_32718_MOESM7_ESM.pdf]

nature portfolio

Corresponding author(s): Anna Katharina Simon

Last updated by author(s): Jul 13, 2022

Reporting Summary

Nature Portfolio wishes to improve the reproducibility of the work that we publish. This form provides structure for consistency and transparency in reporting. For further information on Nature Portfolio policies, see our Editorial Policies and the Editorial Policy Checklist.

Statistics

For all statistical analyses, confirm that the following items are present in the figure legend, table legend, main text, or Methods section.

n/a	Confirmed	
☐	☒	The exact sample size (n) for each experimental group/condition, given as a discrete number and unit of measurement
☐	☒	A statement on whether measurements were taken from distinct samples or whether the same sample was measured repeatedly
☐	☒	The statistical test(s) used AND whether they are one- or two-sided<br>*Only common tests should be described solely by name; describe more complex techniques in the Methods section.*
☒	☐	A description of all covariates tested
☐	☒	A description of any assumptions or corrections, such as tests of normality and adjustment for multiple comparisons
☐	☒	A full description of the statistical parameters including central tendency (e.g. means) or other basic estimates (e.g. regression coefficient) AND variation (e.g. standard deviation) or associated estimates of uncertainty (e.g. confidence intervals)
☐	☒	For null hypothesis testing, the test statistic (e.g. F, t, r) with confidence intervals, effect sizes, degrees of freedom and P value noted<br>*Give P values as exact values whenever suitable.*
☒	☐	For Bayesian analysis, information on the choice of priors and Markov chain Monte Carlo settings
☒	☐	For hierarchical and complex designs, identification of the appropriate level for tests and full reporting of outcomes
☒	☐	Estimates of effect sizes (e.g. Cohen's d, Pearson's r), indicating how they were calculated

Our web collection on statistics for biologists contains articles on many of the points above.

Software and code

Policy information about availability of computer code

Data collection	Flow cytometry data were collected with LSRFortessa™ X-20 Cell Analyzer (BD Biosciences) or LSR II Flow Cytometer (BD Biosciences). Confocal microscopy data were collected with Zeiss LSM 980 confocal microscope 63x oil immersion-lens (Zeiss) or Olympus IX83 inverted microscope equipped with a TIRF module (Olympus). Electron microscopy data were acquired on a JEM 1400plus (JEOL). Western blot data were collected with Odyssey CLx Imaging System (Licor). Proteomics data were generated with Q-Exactive and Orbitrap Fusion Lumos (both Thermo Fisher), The ViiA 7 Real-Time PCR System (Thermo Fisher) was used to conduct the quantitative PCR.
Data analysis	Flow cytometry data were analyzed by Flowjo (version 9.9.6 or 10.7.1, TreeStar). Confocal microscopy analysis was performed using Zeiss ZEN (blue edition) (version 3.3, Carl Zeiss Microscopy GmbH) and ImageJ (version 1.52, NIH) software. Electron microscopy analysis was performed with Fiji software. Western blot data were analyzed by Image Studio Lite (version 5.0, Licor). Proteomics data were analyzed MaxQuant (Andromeda) (version 1.6.10.43) and Python programming (version 3.7.6). Data were exported into Graphpad prism (version 8.2.1) for statistical analysis. Source code for analysing mass spectrometry data is deposited at Github repository: https://github.com/dzhou93/proximity_labelling_pipeline/commit/2e825476556087ae0cff51310556adb278a83d77 (doi:10.5281/zenodo.6792680).

For manuscripts utilizing custom algorithms or software that are central to the research but not yet described in published literature, software must be made available to editors and reviewers. We strongly encourage code deposition in a community repository (e.g. GitHub). See the Nature Portfolio guidelines for submitting code & software for further information.

Data

Policy information about availability of data

All manuscripts must include a data availability statement. This statement should provide the following information, where applicable:

- Accession codes, unique identifiers, or web links for publicly available datasets
- A description of any restrictions on data availability
- For clinical datasets or third party data, please ensure that the statement adheres to our policy

The mass spectrometry data generated in this study have been deposited in the Proteomics Identifications database under accession code PXD033733. UniProt Mus musculus proteome was downloaded from Uniprot database retrieved on retrieved 30/03/2020 (https://www.uniprot.org/proteomes/UP000000589). The raw numbers for charts and graphs are available in the Source Data file whenever possible. Source data are provided with this paper.

Field-specific reporting

Please select the one below that is the best fit for your research. If you are not sure, read the appropriate sections before making your selection.

[x] Life sciences [ ] Behavioural & social sciences [ ] Ecological, evolutionary & environmental sciences

For a reference copy of the document with all sections, see nature.com/documents/nr-reporting-summary-flat.pdf

Life sciences study design

All studies must disclose on these points even when the disclosure is negative.

Sample size	Sample size was determined by the authors based on power calculations. The exact n numbers used in each experiment are indicated in the figure legends.
Data exclusions	For statistical analysis of confocal microscopy, apoptotic and dead cells with abnormal nuclear staining were excluded, which was pre-established.
Replication	Each experiment was repeated at least twice independently with similar results. The information of replication is clearly found in the Figure legends. All attempts of replication were successful. The cell number titrating experiment (Sup Fig. 2f) was only performed once since we want to have an idea of the sensitivity of the protocol, in the meantime, it shows similar a result to the imMEF experiments in Figure 2g.
Randomization	Recipient mice were randomly distributed amongst groups at the start of each experiment. In experiments using cells from mice, littermates with different genotypes from both sexes were randomly assigned to experimental and control groups. In experiments using cell lines, different plates of cells with same confluency were randomly administrated with drugs or vehicles.
Blinding	Blinding was carried out during the software analysis step in all experiments, including flow cytometry, immunofluorescence, quantitative PCR, proteomics, and western blotting. Mouse injection was done blindly by the animal facility staff with no knowledge of genotypes or phenotypes.

Reporting for specific materials, systems and methods

We require information from authors about some types of materials, experimental systems and methods used in many studies. Here, indicate whether each material, system or method listed is relevant to your study. If you are not sure if a list item applies to your research, read the appropriate section before selecting a response.

Materials & experimental systems

n/a	Involved in the study	
[ ]	[x]	Antibodies
[x]	[ ]	Eukaryotic cell lines
[x]	[ ]	Palaeontology and archaeology
[ ]	[x]	Animals and other organisms
[x]	[ ]	Human research participants
[x]	[ ]	Clinical data
[x]	[ ]	Dual use research of concern

Methods

n/a	Involved in the study	
[x]	[ ]	ChIP-seq
[ ]	[x]	Flow cytometry
[x]	[ ]	MRI-based neuroimaging

Antibodies

Antibodies used	1. BioLegend:<br>α-CD4 BV605 GK1.5 (1/400); cat. number 100451; validation: staining on C57BL/6 mouse splenocytes<br>α-CD3 AF700 17A2 (1/200); cat. number 100216; validation: staining on C57BL/6 mouse splenocytes

α-mouse CD3ε biotin 2C11; cat. number 100304
α-CD25 PerCP-Cy5.5 PC61 (1/200); cat. number 102030; validation: staining on C57BL/6 mouse splenocytes
α-CD44 BV785 IM7 (1/400); cat. number 103041; validation: staining on C57BL/6 mouse splenocytes
α-CD45.2 FITC 104 (1/100); cat. number 109806; validation: staining on C57BL/6 mouse splenocytes

α-CD62L APC-Cy7 MEL-14 (1/400); cat. number 104428; validation: staining on C57BL/6 mouse splenocytes
α-CD69 PE-Cy7 H1.2F3 (1/400); cat. number 104512; validation: staining on C57BL/6 mouse splenocytes
α-CD127 (IL-7Rα) PE A7R34 (1/200 for surface staining; 1/100 for intracellular staining); cat. number 135009; validation: staining on C57BL/6 mouse splenocytes
α-CD44 BV785 IM7 (1/400); cat. number 103041; validation: staining on C57BL/6 mouse splenocytes
streptavidin AF647 (1/1,000); cat. number 405237; validation: staining on proximity labelled immortalised MEF cells made from Lc3b-AP2 mice
α-CD107a (LAMP1) FITC 1D4B (1/100); cat. number 121605; validation: staining on C57BL/6 mouse splenocytes
α-CD127 (IL-7Rα) A7R34 (1/100); cat. number 135002; validation: staining on C57BL/6 mouse splenocytes
α-CD25 (IL-2Rα) 7D4/CD25 (1/100); cat. number 154202; validation: staining on C57BL/6 mouse splenocytes
α-CD132 (γc) TUGm2 (1/100); cat. number 132307; validation: staining on C57BL/6 mouse splenocytes

2. BD Biosciences:
α-CD122 (IL-2Rβ) BV650 5H4 (1/200), cat. number 740509; staining on C57BL/6 mouse splenocytes
α-CD132 (γc) BV650 TUGm2 (1/200); cat. number 742573; staining on C57BL/6 mouse splenocytes

3. eBioscience:
α-CD4 PE-Cy7 GK1.5 (1/800), cat. number 25-0041-82; validation: staining on C57BL/6 mouse splenocytes
α-TCR Va2 APC B20.1 (1/400); cat. number 17-5812-82; validation: staining on OT-II mouse splenocytes
α-CD44 PE-Cy7 IM7 (1/200), cat. number 25-0441-82; validation: staining on C57BL/6 mouse splenocytes
α-CD8a FITC 53-6.7 (1/400); cat. number 11-0081-82; validation: staining on C57BL/6 mouse splenocytes
α-phospho-S6 (Ser235, S236) APC cupk43k (1/50); cat. number 17-9007-42; validation: staining on C57BL/6 mouse splenocytes treated with Torin1

4. Cell Signaling Technology:
α-phosphor-Stat5 (Tyr694) AF647 C71E5 (1/100); cat. number 9365S; validation: staining on C57BL/6 mouse splenocytes treated with IL-7
α-β-actin 8H10D10 (1/5,000); cat. number 3700S; validation: C57BL/6 mouse splenocytes lysate
α-LC3B (1/1,000); cat. number 2775S; validation: lysates from C57BL/6 mouse splenocytes treated with Bafilomycin A1
α-c-Myc D84C12 (1:200); cat. number 5605T; validation: staining on C57BL/6 mouse activated T cells

5. from Luminex:
α-LC3 FITC 4E12 (1/20); cat. number FCCH100171; validation: staining on C57BL/6 mouse splenocytes treated with Bafilomycin A1

6. Sigma
α-WIPI2 (1/100); cat. number SAB4200399; validation: staining on C57BL/6 mouse splenocytes

7. Abcam
α-ATG9A EPR2450(2) (1/1,000); cat. number ab108338; validation: C57BL/6 mouse splenocytes lysate

8. MBL International
α-LC3 (1/100); cat. number PM036; validation: staining on C57BL/6 mouse splenocytes treated with Bafilomycin A1

9. Millipore
α-GAPDH 6C5 (1/10,000); cat. number MAB374; validation: C57BL/6 mouse splenocytes lysate

10. Licor
IRDye 680LT Goat anti-Mouse IgG (H + L) (1/7,500); cat. number 926-68070
IRDye 680LT Goat anti-Rat IgG (H + L) (1/10,000); cat. number 926-32219
IRDye 800CW Goat anti-Rabbit IgG (H + L) (1/10,000); cat. number 926-32211
IRDye 680RD Streptavidin (1/1,000); cat. number 926-68079; validation: staining on proximity labelled immortalised MEF cells made from Lc3b-AP2 mice

11. Custom made
α-APEX2 IgG2A (1/200), from Regina Feederle; validation: staining and western-blotting on wild-type or Lc3-AP2 C57BL/6 mouse splenocytes

12. Invitrogen
LIVE/DEAD Fixable Aqua Stain Kit (1/400); cat. number L34957
LIVE/DEAD Fixable Near-IR Stain Kit (1/1,000); cat. number L34975
CellTrace Violet Cell Proliferation Kit (5 μM); cat. number C34557

Validation	Validation method is indicated in the table above. Some antibodies were validated by the manufacturer and information was extracted from their website or documents as follow:<br><br>IRDye® 680LT Goat anti-Mouse IgG Secondary Antibody:<br>Western blot detection of ERK2 in Hela lysate (5 µg – 78 ng). Samples loaded in 4X protein loading buffer (P/N 928-40004), resolved on a 10% Bis-Tris gel, and transferred to Odyssey® nitrocellulose (P/N 926-31090). The membrane was probed with mouse anti-ERK2 (Santa Cruz P/N sc-1647) followed by detection with IRDye 680LT Goat anti-Mouse IgG (P/N 926-68020). (https://www.licor.com/bio/reagents/irdye-680lt-goat-anti-mouse-igg-secondary-antibody)<br><br>IRDye® 680LT Goat anti-Rat IgG Secondary Antibody:<br>Western blot detection of tubulin in C32 lysate. Samples loaded in 4X Protein Sample Loading Buffer (P/N 928-40004), resolved on a 10% Bis-Tris gel, and transferred to Odyssey® nitrocellulose (P/N 926-31090). The membrane was blocked with Odyssey Blocking Buffer (P/N 927-40000) and probed with rat anti-tubulin (Novus Biologicals P/N NB600-506) followed by detection with IRDye 680LT Goat anti-Rat IgG (P/N 926-68029). (https://www.licor.com/bio/reagents/irdye-680lt-goat-anti-rat-igg-secondary-antibody)<br><br>IRDye® 800CW Goat Anti-Rabbit IgG:<br>This antibody was tested by Dot Blot and/or solid-phase adsorbed for minimal cross-reactivity with human, mouse, rat, sheep, and chicken serum proteins. The conjugate has been specifically tested and qualified for Western blot and In-Cell Western Assay applications. (https://www.licor.com/documents/rfm2hw40wf33p06f3ndjrcorwi5usbft)<br><br>Both LIVE/DEAD™ Fixable Near-IR Dead Cell Stain Kit and LIVE/DEAD™ Fixable Aqua Dead Cell Stain Kit:<br>Validation was performed through staining a mixture of live and heat-treated Jurkat cells. The live cell population is easily distinguished from the killed population, and nearly identical results were obtained using unfixed cells. (https://www.thermofisher.com/document-connect/document-connect.html?url=https://assets.thermofisher.com/TFS-Assets%2FLSG%2Fmanuals%2Flive_dead_fixable_dead_cell_stains_man.pdf)<br><br>CellTrace™ Violet:<br>Validation was performed on human peripheral blood mononuclear cells isolated from whole blood, stimulated to proliferate with 200 ng mouse anti-human CD3 antibody (clone S4.1) and 100 ng Interleukin-2 (IL-2) per milliliter cells and incubated at 37°C and 5% CO2 for 7 days.(chrome-extension://efaidnbmnnnibpcajpcglclefindmkaj/https://www.thermofisher.com/content/dam/LifeTech/migration/en/filelibrary/cell-tissue-analysis/pdfs-posters-for-cta.par.38129.file.dat/celltrace-advanced-techniques.pdf)

Animals and other organisms

Policy information about studies involving animals; ARRIVE guidelines recommended for reporting animal research

Laboratory animals	Atg7flox/flox mice (from M Komatsu) and CD4cre mice (from Adeline Hajjar) were crossed to obtain CD4cre Atg7-/- mice (T-Atg7-/-) on a C57BL/6 background. All mice were 6-8 weeks of age at the start of each experiment and were matched in age and gender. CD4cre Atg7+/+ littermates were used as wild-type controls (wild-type). C57BL/6 SJL CD45.1 mice for bone marrow chimera and T cell adoptive transfer were purchased from Charles River, UK. OT-II mice were crossed with CD4cre Atg7-/- mice. CD4cre Atg16l1-/- mice were from Kevin Maloy. Lc3b-APEX2 (Lc3b-AP2) mice were generated by Cyagen, by constitutively knocking in an APEX-tag and a GGGGSGGGGGS-linker into the exon 1 of the mouse Map1lc3b allele.<br><br>All mice were held in Biological Support Unit at the Kennedy Institute of Rheumatology. All mice were under specific pathogen-free level maintenance at 24 °C, 50% humidity, and a 12:12 h light/dark cycle. Mice were kept in individually ventilated cages, with ad libitum access to autoclaved water and irradiated food pellets. Cages were changed once weekly, and the health status of the mice was monitored based on body weight, coat, and behaviour. Littermates from both sexes were randomly assigned to experimental groups. The exact number of the mice used for the experiments are indicated for each experiment in the figure legends.
Wild animals	The study did not involve wild animals.
Field-collected samples	The study did not involve samples collected from the field.
Ethics oversight	Animal experiments were performed in accordance with institutional policies, British federal regulations, and were approved by UK Home Office (project license PPL 30/3388).

Note that full information on the approval of the study protocol must also be provided in the manuscript.

Flow Cytometry

Plots

Confirm that:

- [x] The axis labels state the marker and fluorochrome used (e.g. CD4-FITC).
- [x] The axis scales are clearly visible. Include numbers along axes only for bottom left plot of group (a 'group' is an analysis of identical markers).
- [x] All plots are contour plots with outliers or pseudocolor plots.
- [x] A numerical value for number of cells or percentage (with statistics) is provided.

Methodology

Sample preparation	Blood samples were obtained from the tail vein. Spleens or lungs were obtained directly from mice. Single cell splenocytes were prepared by meshing whole spleens through 40 µm strainers (BD Biosciences) using a syringe plunger. Identification of viable cells was done by Fixable Near-IR or Fixable Aqua dead cell staining (Invitrogen). Erythrocytes were lysed by ACK lysis buffer treatment for 5 min at room temperature. Surface staining was performed for 20 min at 4°C. For intracellular staining, cells were fixed with Fixation Buffer (BioLegend) in the dark for 20 min at room temperature after surface staining, then permeabilised with Intracellular Staining Perm Wash Buffer (BioLegend) and incubated with antibody diluted in Perm/Wash Buffer in the dark for 1 hour at room temperature. To measure cell proliferation profile in vitro, cells isolated from mouse spleens were stained with CellTrace Violet (5 µM, Invitrogen) for 20 min at room temperature in dark. Tetramer staining was performed through incubation with 20ug/ml tetramer in PBS at 37 °C for 30 min before surface staining. Phospho-Stat5 staining was performed through fixation with pre-heated 2% PFA for 15 min at 37 °C after the live-dead staining, permeabilised with pre-cooled 90% methanol for more than 4 hours, and stained overnight with the antibody diluted with 0.5% BSA in PBS at 4 °C overnight. To measure the autophagic flux, cells were treated with Bafilomycin A1 (10 nM) or DMSO for 2 hours before staining. We adapted the Guava Autophagy LC3 Antibody-based Detection Kit (Luminex) as follows: After surface staining, cells were washed once with Assay Buffer. After permeabilization with 0.05% Saponin, cells were spun immediately and incubated with anti-LC3 (FITC) antibody (1:20 diluted in Assay Buffer) at room temperature for 30 minutes in dark. After extensive washing with Assay Buffer, cells were fixed with 2% PFA and underwent flow cytometry analysis. Autophagic flux was calculated as LC3-II mean fluorescence intensity of (BafA1-Vehicle)/Vehicle. All samples were washed and stored in PBS containing 2% FBS (Sigma-Aldrich) and 5mM of EDTA (Sigma-Aldrich) before acquisition.
Instrument	Multi-parametric flow cytometric analysis was performed using LSRFortessa™ X-20 Cell Analyzer (BD Biosciences) or LSR II Flow Cytometer (BD Biosciences).
Software	Flow cytometry data were analyzed by Flowjo (Tree Star v9.9.6 or v10.8.1)
Cell population abundance	For bulk CD4 assays, CD4+ T cells were isolated from spleens of Lc3-AP2 or T-Atg7-/- mice using the EasySepTM Mouse CD4+ T cell Isolation Kit (StemCell Technologies). For analysis of specific cell subsets and CD4+ T cell enrichment, erythrocytes were lysed and remaining splenocytes were incubated with α-B220 biotin (RA3-6B2, BioLegend), and α-CD8a biotin (53-6.7, BioLegend) antibodies for 20 min at room temperature, followed by incubation with EasySep™ Streptavidin RapidSpheres (StemCell Technologies) for 5 min at room temperature, and further magnetic separation. After enrichment, cells were then stained for phenotypical markers, and subpopulations of interest were sorted on a FACS Aria cell sorter (BD Biosciences). Post-sort fractions were also acquired directly after sorting to check the purity. Flow cytometry analysis of adoptive transfer experiments was never limited by cell numbers of target cells.
Gating strategy	For all samples the following gating strategy was used: lymphocytes (SSC-A/FSC-A), exclusion of doublets (SSC-A/SSC-H), live cells (SSC-A/Live/Dead marker Near-IR or Aqua). To identify congenitally marked CD4+ T cells in bone marrow chimaera experiment, cells were gated for CD19, CD4 and for the congenic markers CD45.2 or CD45.1. To identify congenitally marked OT-II TCR transgenic CD4+ T cells in adoptive transfer experiment, cells were gated for CD4, TCR Vα2 (OT-II) and for the congenic marker CD45.2. Non-activated CD4+ T cell subsets were gated on CD62L and CD44 (naïve CD4+ T cell - CD62L-positive & CD44-negative; effector CD4+ T cell - CD62L-negative CD44-positive), while activated CD4+ T cells were gated on CD25-positive population.

- [x] Tick this box to confirm that a figure exemplifying the gating strategy is provided in the Supplementary Information.
